# Characterization of alpha-1-acid glycoprotein as a potential biomarker for breast cancer

**DOI:** 10.1080/21655979.2022.2036303

**Published:** 2022-02-21

**Authors:** Luo Qiong, Jun Yin

**Affiliations:** Department of Breast and Thyroid Surgery, Affiliated Hengyang Hospital, Southern Medical University (Hengyang Central Hospital), Hengyang, Hunan, China

**Keywords:** Breast cancer, immunohistochemistry, alpha-1-acid glycoprotein, biomarker

## Abstract

Breast cancer is a malignant tumor that poses a serious threat to the health of women worldwide. The early diagnosis of patients with cancer or those at high risk remains difficult, which makes treatment challenging. Therefore, the study of diagnostic biomarkers for early detection of cancer is very important. AGP biomarkers are expected to be potential biomarkers for early detection of breast cancer. This study aimed to explore the potential of alpha-1-acid glycoprotein (AGP) as a biomarker for the diagnosis of breast cancer. The results revealed that the expression of AGP was high in breast cancer cells and tissues and was higher at stage IV than at stages III and II. Knockdown of *ORM1*, which encodes AGP, in MCF-7 cells suppressed the production of the inflammatory factors interleukin *(IL)-1β, IL-8*, and tumor necrosis factor-α. These results suggest that AGP can serve as a therapeutic target and/or diagnostic marker for breast cancer. Overall, we found that AGP can serve as a biomarker for breast cancer and inhibit secretion of related pro-inflammatory facto by blocking ORM1 expression.

## Graphical Abstract

**Figure:**
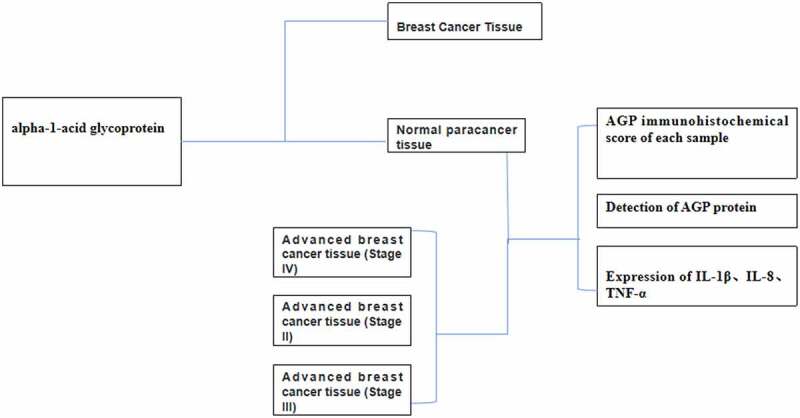

## Introduction

Breast cancer is among the most common malignancies in women [1]. It accounts for approximately one-fourth of all cancer cases in women, and the mortality rate is >15% [2,3]. Therefore, breast cancer poses a serious threat to women’s health [4]. The successful and early screening of breast cancer is hampered by the lack of biochemical markers. Commonly used imaging techniques include mammography and high-frequency color Doppler ultrasound, both of which have advantages and disadvantages, although they are often used in combination to avoid misdiagnosis [5]. Recent studies have shown that many new proteins, such as dermcidin, are involved in tumor formation and metastasis and are directly associated with tumor development [6,7]. These proteins may be utilized as a biomarker for the early diagnosis of tumors.

Biomarkers, which are biological components that are present in tissues or body fluids, are used in clinical diagnosis, prognosis, care, and risk assessment [8,9]. Biomarkers typically vary in content and expression according to the degree of disease progression [10,11]; this suggests that they can be used to assess disease development or treatment response. However, owing to the complexity of carcinogenesis and the specific reliability of biomarkers, not many biomarkers can be used to accurately diagnose breast cancer. To date, only MUC-1 and carcinoembryonic antigen have been officially adopted for use in the diagnosis of breast cancer [12–14]. Therefore, there is a need to identify additional biomarkers for the accurate diagnosis of breast cancer.

Alpha-1-acid glycoprotein (AGP), also known as orosomucoid (ORM), is a nonspecific acute reactive protein present in the blood stream and is encoded by *ORM1* and *ORM2*. It is considered to be a natural anti-inflammatory and immune modulator [15]. Alterations in the protein levels of AGP have been well documented for numerous physiological and pathophysiological conditions including lung and breast cancer [16] and malignant mesothelioma [17]. It has been suggested that a marked increase in AGP concentration may limit adverse reactions such as inflammation by providing a form of negative feedback [18]. In terms of plasma concentration, increased levels of AGP have been detected in the plasma of patients with breast cancer [19] and have also been shown to increase with disease progression [20]. This indicates that the prognosis of breast cancer appears to be linked to the AGP levels in particular. Expression of the gene encoding AGP is controlled by a combination of major regulatory mediators, glucocorticoids, and a cytokine network involving interleukin (IL)-1-beta (IL-1β), tumor necrosis factor-alpha (*TNF-α), IL-6*, and IL-6-related cytokines.

Further studies are needed on the reliability and effectiveness of AGP biomarkers in clinical application. Therefore, this study detected the expression of AGP in the cells and tissues of breast cancer patients and blocked the expression of ORM1 to inhibit the secretion of related pro-inflammatory factors. To prove the potential of AGP as a biomarker for early detection of breast cancer will provide a new idea for clinical treatment of breast cancer.

## Materials and methods

### Tissues

Tumor tissues and normal appearing tissues were obtained from 90 patients with breast cancer from January 2019 to October 2019 at the Department of Breast and Thyroid Surgery of the Southern Medical University (Hengyang Central Hospital). Based on the diagnostic results, the patients were categorized into early-, middle-, and late-stage disease, with 30 patients with early-stage (stage II), 30 with middle-stage (stage III), and 30 with late-stage (stage IV). All the patients were diagnosed with breast cancer before or after surgery, and no other cancer was evident. Patients who had undergone preoperative radiotherapy, chemotherapy, targeted treatment, or immunotherapy were excluded from this study.

### Biochemical reagents

We used an AGP1 antibody (Genway; San Diego, CA, USA), enzyme-linked immunosorbent assay (ELISA) kits, Trizol reagent (Invitrogen, Grand Island, NY, USA), and PrimeScript RT Master Mix Perfect Real-Time Kit (Takara, Otsu, Japan). Real-time quantitative RT-PCR analysis was performed using SYBR RT-PCR kits (Takara).

### Cell culture and transfection

The breast cancer cell line MCF-7 and normal breast epithelial cell line MCF-10A were purchased from the American Type Culture Collection. The cells were cultured in Dulbecco’s modified Eagle’s medium containing 10% fetal bovine serum and 10 μg/ml insulin for 24 h at 37°C and 5% CO_2_. Then, MCF-7 cells were inoculated into a 12-well plate at a density of 2 × 10^5^ cells/well, and each group was arranged in triplicate wells. siRNA-ORM1 was transfected with Lipofectamine ^TM^ 2000 reagent (Thermo Scientific, USA). The experiment was divided into an MCF-10 group (control), MCF-7 group (MCF-7), and siRNA-ORM1-transfected MCF-7 group (MCF-7+ si-ORM1).

### Immunohistochemistry

The surfactant protein method was employed to detect the immunohistochemical expression of AGP. The same cancer cells were used as the negative control and experimental groups. The cancerous tissue and normal appearing tissues were fixed in 10% methanol, embedded in paraffin for 24 h, and then cut into sections. The tissue sections were dewaxed and hydrated. Endogenous peroxidase was inactivated by treating the sections with 3% H_2_O_2_ at room temperature in a wet box for 10 min, followed by washing with phosphate-buffered saline (PBS). Then, 30 μl of 5% bovine serum albumin (BSA) was added, followed by incubation for 30 min at room temperature. The experimental group was incubated with 30 μl of rabbit anti-AGP1 antibody (Abcam) (1:200 dilution), and the control group was incubated with the same amount of 5% BSA at 4°C overnight. The next day, both groups were incubated with 30 μl of secondary antibody (Abcam) at room temperature for 30 min and then washed with PBS. Next, 30 μl of streptavidin-biotin peroxidase solution was added, followed by incubation at room temperature for 30 min. Finally, 3, 3′ diaminobenzidine tetrahydrochloride reagent was added for 5 min and the slides were stained with hematoxylin. After dehydration with graded concentrations of ethanol, the tissue sections were sealed using neutral resin for observation.

IHC Profiler was used to analyze the staining, and the staining intensity and staining area of the positive cells were scored using a scale. The number of positive cells was graded as 0 (<5%), 1 (5%–25%), 2 (25%–50%), 3 (51%–75%), or 4 (>75%). The two grades were added together, and the specimens were assigned to one of four score categories: 0–1 (-), 2 (+), 3–4 (++), and greater than 5 (+++). The positive expression rate was expressed as the percent of the addition of (++) and (+++) to the total number.

### Western blot analysis

Approximately 0.1 g of tissue samples was excised from tumor tissues and normal appearing tissues. The samples were cut into sections and placed in a glass homogenizer. Phenylmethylsulfonyl fluoride lysate (1:10) buffer was added, and the samples were homogenized on ice for 30 min. The supernatant was obtained after centrifugation. The cells were collected and placed on ice 24 h after culture, and RIPA lysis buffer (50 mM Tris-HCl, pH 7.4, 150 mM NaCl, 1% NP-40, and 0.1% SDS) was added to lyse the cells. The total protein concentration was measured using the bicinchoninic acid (Sigma) method. After determining the protein concentration, the protein was mixed with the buffer solution and heated until denaturation. Sodium dodecyl sulfate–polyacrylamide gel electrophoresis was employed to separate the proteins, followed by transfer to a polyvinylidene difluoride membrane. The membrane was immersed in a blocking solution for 1–2 h at 37°C. Then, the membrane was incubated with rabbit anti-AGP antibody (Abcam) overnight at 4°C and washed three times with PBS. Next, the membrane was incubated with a secondary antibody (Abcam) for 2 h at 37°C. After washing the membrane with PBS, the protein bands were visualized using chemiluminescence. ImageJ software was used to examine the band intensities, and relative protein expression was determined by calculating the ratio of the sample to the internal reference. Protein expression in each group was normalized to that of β-actin.

#### *Determination of* IL-1β, IL-8, *and* TNF-α *levels using ELISA*

Tumor tissues and normal appearing tissues were washed with 5–10 ml of precooled PBS (mass:volume ratio of tissue to PBS of 1:5) and subject to grinding. The grinding process was performed on ice. After grinding, the tissue homogenates were centrifuged for 5 min (5000 × g). For cell samples, the collected cells were washed with precooled PBS and disrupted via ultrasonication. After centrifugation (10 min, 1500 × g), the supernatant was collected for detection. The cell and tissue supernatants were evaluated using double antibody-sandwich ELISA. The expression levels of *TNF-α, IL-1β*, and *IL-8* in the supernatant were determined following the ELISA kit instructions. Each group was tested in triplicate.

### Quantitative RT-PCR

Approximately 100 mg of tumor tissues and normal appearing tissues were collected and fully ground in liquid nitrogen. Total RNA was extracted using Trizol reagent. Reverse transcription and amplification were performed according to the PCR kit instructions. Quantitative PCR was conducted using the SYBR green method. Each group was measured in triplicate. The housekeeping gene, *GAPDH*, was used as a reference gene. Based on the CT value of each sample, the relative expression level was determined using the 2^−ΔΔCt^ method.

### Statistical analysis

SPSS Statistics for Windows, version 17.0 (SPSS Inc., Chicago, IL, USA) was used for data analysis. The results were presented as the mean ± standard deviation, Chi-square test was used for comparison between groups, and P < 0.05 was considered statistically significant.

## Results

### AGP expression in breast cancer tissues and normal appearing tissues

**Figure 1.**
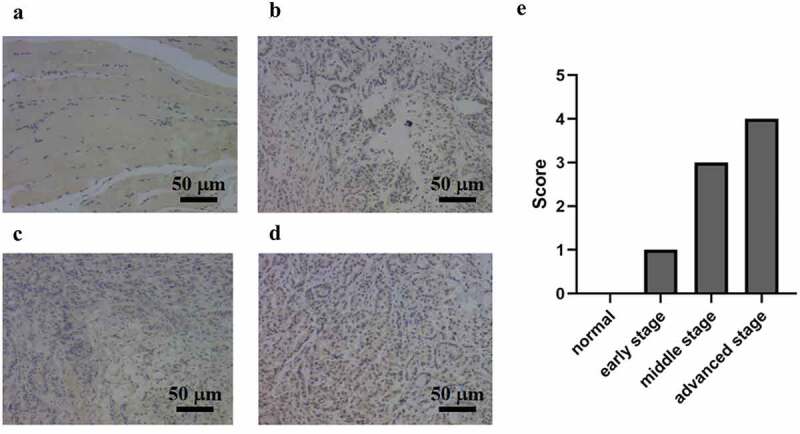
**AGP expression at different stages of breast cancer in tumor tissues and adjacent normal tissues detected using immunohistochemistry (×200)**. (a) normal adjacent tissue. (b) early-stage breast cancer tissue. (c) middle-stage breast cancer tissue. (d) late-stage breast cancer tissue. (e) scoring of immunohistochemistry results for AGP in each sample.

**Figure 2.**
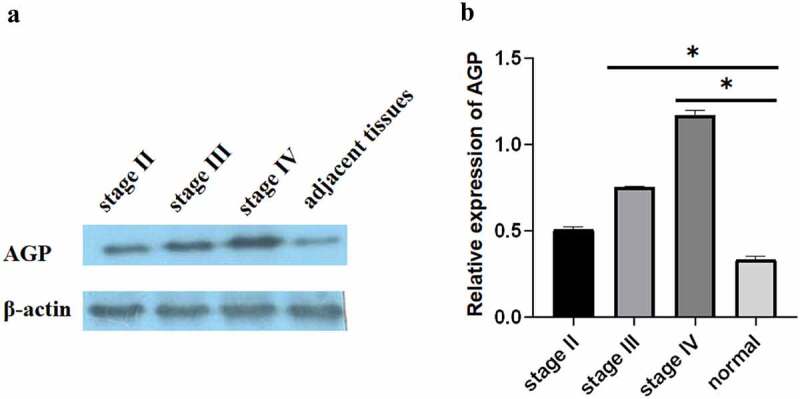
**Expression of AGP in breast cancer tissues and adjacent normal tissues using Western blot analysis**. (a-b). Western blot analysis was performed to evaluate AGP protein expression at different stages of breast cancer: early-stage (stage II), middle-stage (stage III), late-stage (stage IV), and adjacent normal tissue. The relative expression of AGP in stage III and stage IV was significantly higher than that in normal tissues (*P < 0.05).

Compared with that in normal appearing tissues, the immunohistochemistry results showed that the AGP staining areas in the late-, middle-, and early-stage cancer tissues were higher and that the staining intensity was increased by approximately 15–30-fold [Figure 1]. The scores for the normal, early, middle, and advanced stages were 0, 1, 3, and 4. In particular, the expression level of AGP was also increased with the development of cancer and was higher at the late stage compared with that at the middle stage and higher at the middle stage compared with that at the early stage. Similar results were obtained using Western blot analysis [Figure 2]. Quantitative analysis with ImageJ revealed that AGP gene expression was significantly higher in tumor tissues compared with that in normal appearing tissues. Furthermore, the expression increased as the cancer progressed (P < 0.05). These results indicate that the AGP expression levels are increased in cancer and increase with the progression of cancer.

### Expression of IL-1 β, IL-8, TNF-α and AGP in McF-10, McF-7 and McF-7 + Si-ORM1 in breast cancer tissues

**Figure 3.**
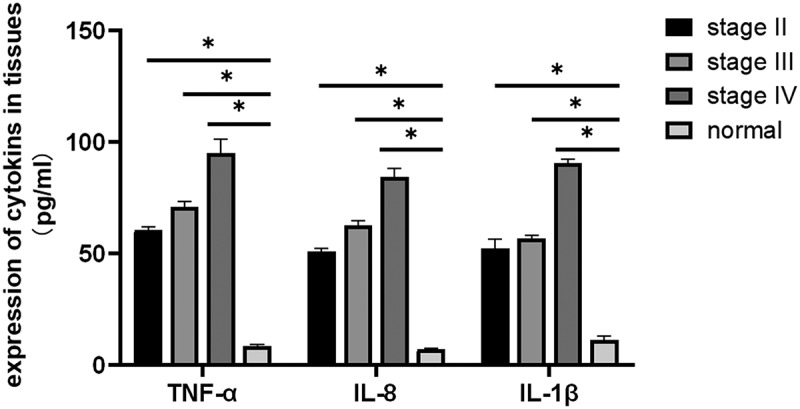
**Determination of the expression levels of *IL-1β, IL-8*, and *TNF-α* in breast cancer tissues and adjacent normal tissues using ELISA (*P* < 0.05)**. Compared with those in normal adjacent tissues, *TNF-α, IL-1 β*, and *IL-8* expression levels were significantly different at all cancer stages: early-stage (stage II), middle-stage (stage III), and late-stage (stage IV) (*P < 0.05).

**Figure 4.**
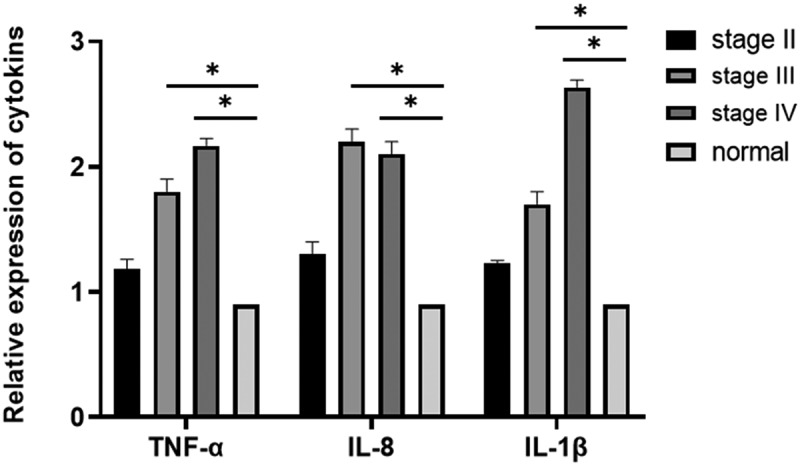
**Determination of the expression levels of IL-1β, IL-8, and TNF-α in breast cancer tissues and adjacent normal tissues using qRT-PCR (P < 0.05)**. Relative expression levels of *TNF-α, IL-8*, and *IL-1β* in middle-stage (stage III) and late-stage (stage IV) breast cancer compared with those in normal adjacent tissues (*P < 0.05).

The inflammatory factors *IL-1β, IL-8*, and *TNF-α* can alter the tumor microenvironment and promote tumor growth, angiogenesis, and metastasis. Therefore, it is of great importance to study the relationship between inflammatory factors (*IL-1β, IL-8, TNF-α*) and breast cancer. ELISA was performed to evaluate the expression levels of *IL-1 β, IL-8*, and *TNF-α* in the tissues. The results showed that *IL-1β, IL-8*, and *TNF-α* expression levels in breast cancer tissues were elevated compared with those in the normal appearing tissues. The expression levels of *IL-1β, IL-8*, and *TNF-α* increased with progression through the clinical stages of breast cancer [Figure 3], and the differences were statistically significant (*P* = 0.03). The expression levels of *IL-1β, IL-8*, and *TNF-α* were significantly elevated in breast cancer tissues compared with that in normal appearing tissues (Figure 4, Table 1). In addition, the expression levels in the cancer tissues increased with increasing stage (stage II, III, and IV) of disease (*P* = 0.035).

**Table 1.** Primer sequences for *qRT-PCR.*

Target Gene	Forward (5’ → 3’)	Reverse (5’ → 3’)
IL-1β	TGCCACCTTTTGACAGTGATG	AAGGTCCACGGGAAAGACAC
IL-8	CTGCAAGAGACTTCCATCCAG	AGTGGTATAGACAGGTCTGTTG
TNF – α	CGCTGAGGTCAATCTGCCCAAGTAC	GGGGGCTGGGTAGAGAATGGATG
ORM1	ACACCACCTACCTGAATGTCC	GTGAGCGAAATGCTCTTGGC
GAPDH	ACAACTTTGGTATCGTGGAAGG	GCCATCACGCCACAGTTTC
siRNA ORM1	GAGGTTGATGTATGTGTAGGTTTCACTCCT	CTTACCCAGCTCAGGGTCTC

### *Overexpression of* ORM1, IL-1β, IL-8, *and* TNF-α *in MCF-7 cells*

**Figure 5.**
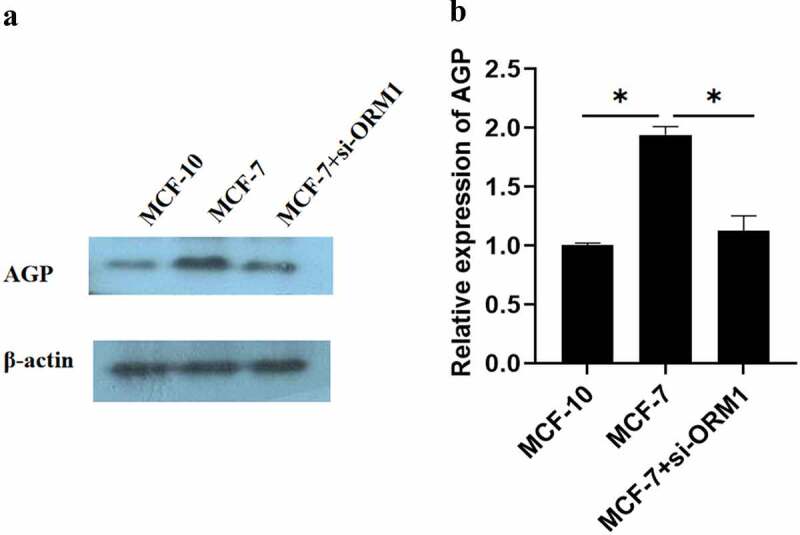
**Expression of AGP in MCF-10, MCF-7, and MCF-7+ si-ORM1 cells determined using Western blot analysis**. (a-b). AGP protein expression in MCF-7 breast cancer cells and cells transfected with si-ORMI were evaluated by Western blotting analysis (*P < 0.05). The experiments were conducted in triplicate.

The results of Western blot revealed that OMR1 protein-induced AGP expression was considerably higher in MCF-7 cells than in the control cells (MCF-10) (P < 0.05) [Figure 5]. However, the production of AGP in MCF-7 cells transfected with si-ORM1 was significantly lower than that in MCF-7 cells (*P* = 0.044).

**Figure 6.**
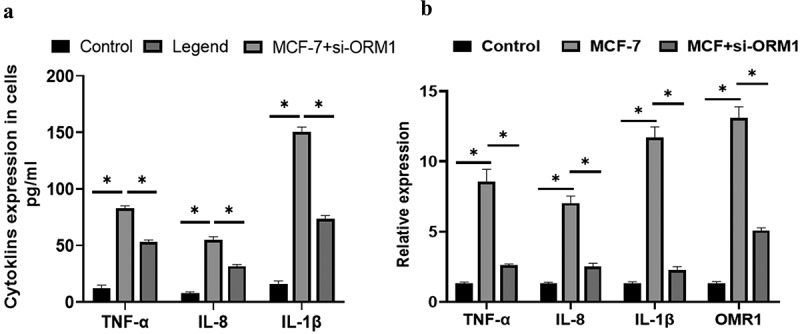
**The expressions of *IL-1β, IL-8, TNF-α* and *ORM1* in breast cancer cells were detected by different methods**. A:The levels of *IL-1β*, IL-8, and *TNF-α* in MCF-7 cells and cells transfected with si-ORM1 were compared. The expression levels of *IL-1β, IL-8*, and *TNF-α* in MCF-7 cells were significantly higher than those in the control group (P < 0.05). MCF-7+ si-ORM1 group suppressed the expression of *ORM1* compared with that in the MCF-7 group (*P < 0.05). B: The relative expression levels of *ORM1, IL-1β, IL-8*, and *TNF-α* under different cell conditions were compared. The expression levels of *TNF-α, IL-8, IL-1β*, and *ORM1* in MCF-7 cells were significantly higher than those in the control group (P < 0.05, σ^2^ = 0.03). The MCF-7+ si-ORM1 group exhibited decreased expression compared with the MCF-7 group (*P < 0.05, σ^2^ = 0.03). The experiments were conducted in triplicate.

The ELISA results revealed that in MCF-7 cells, the secretion of *IL-1 β, IL-8*, and *TNF-α* was markedly higher than that in the control group (Figure 6a) (*P* = 0.044). However, following si-ORM1 treatment, the secretion of these inflammatory factors was considerably lower in si-ORM1-transfected MCF-7 cells than in MCF-7 cell. *ORM1* expression was increased in MCF-7 cells compared with that in the control cells, and there was a significant increase in the expression levels of the proinflammatory cytokines *IL-1β, IL-8*, and *TNF-α* (P < 0.05) (Figure 6b). Compared with that in the MCF-7 group, the expression of ORM1 and the proinflammatory cytokines were significantly decreased in the MCF-7+ si-ORM1 group (P < 0.05). These results showed that the expression levels of *IL-1β, IL-8*, and TNF-α were regulated by inducing *ORM1* gene in breast cancer cells.

### Relationship between AGP expression and clinical characteristics

There was no significant correlation between the expression of AGP in breast cancer tissues and some clinicopathological factors (E-age menopausal bed bath period, tumor size, pathological type, differentiation, lymph node metastasis, etc.), and these differences were not statistically significant (P > 0.05, Table 2).

**Table 2.** Alpha-1 acid glycoprotein (AGP) in 90 cases of breast cancer and its relationship with clinical characteristics

Characteristics of clinical cases	case	High expression	Low expression	χ^2^	*P*
Age				0.022	0.882
≥53	63	53	10		
< 53	27	20	7		
menopause				0.007	0.933
Yes	58	49	9		
No	32	19	8		
Clinical stages				0.072	0.788
I	30	26	4		
II	30	24	6		
III	30	23	7		
Pathological type				0.001	0.978
Invasive ductal carcinoma	39	16	8		
Invasive lobular carcinoma	51	19	9		
Lymph node metastasis				0.022	0.882
yes	34	27	7		
no	56	46	10		

## Discussion

In the present study, the expression of AGP in breast cancer cells and normal breast epithelial cells was analyzed. The results indicated that AGP expression in breast cancer tissues or cells was considerably higher at both the protein and gene levels compared with that in normal cells and tissues. In addition, AGP expression increased at different stages of the disease. Furthermore, the proinflammatory factors *IL-1β, IL-8*, and *TNF-α*, were highly expressed in breast cancer cells and tissues, and they were inhibited in MCF-7 breast cancer cells by downregulating the expression of *ORM1*. Thus, knockdown of *ORM1* in MCF-7 cells inhibited the secretion of proinflammatory factors. The effects of the reduction of AGP expression in a colon cancer cell line, HT29, was reported by Kim et al. and the effects of the reduction of ORM2 expression in HCC cell lines was reported by Fang et al. [21,22]. siORM1 knockdown in MCF-7 cells and its observed effects are consistent with the findings of previous studies. This provides a new therapeutic strategy for cancer treatment. The identification of alterations in AGP glycosylation specific to individual stages of breast cancer could, through being altered to the “normal” healthy AGP glycosylation pattern, result in the development of a diagnostic test based on AGP glycosylation for the onset, progression and/or prognosis of breast cancer.

Increased AGP expression in the tissues of patients with breast cancer may be associated with the levels found in the sera of patients [16,23]. Although the exact physiological or pathological function is unclear, AGP is a potential biomarker that warrants further study. In addition, the different stages of breast cancer were accompanied by changes in the levels of inflammatory factors. Previous and current studies strongly indicate that AGP is synthesized rapidly, reflecting the response of patients to treatment [24]. In this study, the content and expression of AGP in the breast cancer cell and normal breast epithelial cells were analyzed and it was confirmed that AGP expression in breast cancer tissues or cells are considerably higher at both protein and gene levels than that in normal cells and tissues. Further studies should include the measurement of AGP expression in other tumor types as predictive biomarkers for immunotherapy and the analysis of the mechanism of fucosylation that occurs in AGP molecules during treatment [25].

In conclusion, the strength of this study lies in the use of Western blotting, immunohistochemistry, and ELISA to demonstrate the similarities and differences in the expression of AGP in cancer tissues and adjacent normal tissues in patients with breast cancer. increased expression of AGP in breast cancer cells and tissues indicates that AGP can serve as a biomarker for breast cancer. The downregulation of *ORM1* expression in breast cancer cells can inhibit the production of proinflammatory factors, which provides insight into a possible clinical treatment strategy; however, further studies are needed to address these issues.

## Conclusions

The expression of AGP and *IL-1β, IL-8* and *TNF-α* in breast cancer tissues were significantly higher than those in normal appearing tissues. The expression of AGP induced by *OMR1* protein in McF-7 breast cancer cells and the secretion of *IL-1 β, IL-8* and *TNF-α* in McF-7 breast cancer cells were significantly higher than that in normal appearing tissues.There was no significant correlation between AGP expression in breast cancer tissues and e-age menopausal bed bath period, tumor length and pathological differentiation of lymph node metastasis.

## Data Availability

All data are available from the corresponding authors on reasonable request.
